# (1, 3)-β-D-glucan assay for diagnosing invasive fungal infections in critically ill patients with hematological malignancies

**DOI:** 10.18632/oncotarget.7471

**Published:** 2016-02-18

**Authors:** Elie Azoulay, Nicolas Guigue, Michael Darmon, Djamel Mokart, Virginie Lemiale, Achille Kouatchet, Julien Mayaux, François Vincent, Martine Nyunga, Fabrice Bruneel, Antoine Rabbat, Stéphane Bretagne, Christine Lebert, Anne-Pascale Meert, Dominique Benoit, Frédéric Pene

**Affiliations:** ^1^ Medical ICU and Mycology Department, Saint-Louis Hospital, Paris, France; ^2^ Medical-Surgical ICU, Saint-Etienne University Hospital, Saint-Étienne, France; ^3^ Medical-Surgical ICU Paoli Calmette Cancer Institute, Marseille, France; ^4^ Medical ICU, Angers University Hospital, Angers, France; ^5^ Medical ICU CHU Pitié-Salpêtrière, Paris, France; ^6^ Medical-Surgical ICU, Montfermeil Hospital, Montfermeil, France; ^7^ Medical ICU and Pulmonary Department, Cochin Hospital, Paris, France; ^8^ Medical-Surgical ICU, La Roche Sur Yon Hospital, La Roche Sur Yon, France; ^9^ Medical-Surgical ICU, Jules Bordet Cancer Institute, Brussels, Belgium; ^10^ Medical-Surgical ICU, Ghent University Hospital, Gent, Belgium

**Keywords:** invasive fungal infection, intensive care units, diagnostic tests, (1–3)-beta-D-glucan assay

## Abstract

Invasive fungal infections (IFIs) are life-threatening complications of hematological malignancies that must be diagnosed early to allow effective treatment. Few data are available on the performance of serum (1–3)-β-D-glucan (BG) assays for diagnosing IFI in patients with hematological malignancies admitted to the intensive care unit (ICU). In this study, 737 consecutive patients with hematological malignancies admitted to 17 ICUs routinely underwent a BG assay at ICU admission. IFIs were diagnosed using standard criteria applied by three independent specialists. Among the 737 patients, 439 (60%) required mechanical ventilation and 273 (37%) died before hospital discharge. Factors known to alter BG concentrations were identified in most patients. IFIs were documented in 78 (10.6%) patients (invasive pulmonary aspergillosis, *n* = 54; *Pneumocystis jirovecii* pneumonia, *n* = 13; candidemia, *n* = 13; and fusarium infections, *n =* 3). BG concentrations (pg/mL) were higher in patients with than without IFI (144 (77–510) vs. 50 (30–125), < 0.0001). With 80 pg/mL as the cutoff, sensitivity was 72%, specificity 65%, and area-under-the-curve 0.74 (0.68–0.79). Assuming a prevalence of 10%, the negative and positive predictive values were 94% and 21%. By multivariable analysis, factors independently associated with BG > 80 pg/mL were IFI, admission SOFA score, autologous bone-marrow or hematopoietic stem-cell transplantation, and microbiologically documented bacterial infection. In conclusion, in unselected critically ill hematology patients with factors known to affect serum BG, this biomarker showed only moderate diagnostic performance and rarely detected IFI. However, the negative predictive value was high. Studies are needed to assess whether a negative BG test indicates that antifungal de-escalation is safe.

## INTRODUCTION

Advances in the management of patients with hematological malignancies have led to improved survival, but have also increased the incidence of invasive fungal infections (IFIs). [1] despite the widespread use of prophylactic antifungal agents. Early recognition and treatment of life-threatening IFIs in patients with hematological malignancies is crucial [2]. Both cultures and imaging studies often fail to establish the definitive diagnosis of specific IFIs, leading to prolonged empirical antifungal therapy, whose effectiveness may be limited [1, 3].

IFIs carry a grim prognosis in patients with hematological malignancies admitted to the ICU, particularly those receiving mechanical ventilation or having a history of allogeneic bone-marrow or hematopoietic stem-cell transplantation [4]. In these patients, co-infections [5] and organ dysfunctions are common and may impair the performance of diagnostic biomarkers for IFIs [6, 7].

Plasma (1, 3)-β-D-glucan (BG) antigenemia is among the revised criteria for IFI and probable IFI developed by the European Organization for Research and Treatment of Cancer and Mycoses Study Group (EORTC-MSG) [1, 8]. BG is a cell-wall component of most fungi responsible for IFIs, except *Zygomycetes* spp. and *Cryptococcus* spp. The assay relies on activation by BG of factor G in the coagulation cascade in the Limulus amebocyte lysate, which leads to quantifiable transformation of a chromogenic substrate [9]. BG has been evaluated using various study designs, in different settings. In case-control studies comparing patients with proven or probable IFI to healthy controls or patient populations at low risk for IFI, BG assay was 50%–90% sensitive and 70%–100% specific [10–13]. Performance was better in prospective studies of homogeneous populations at high risk for IFI [14, 15] but differed markedly between patients with acute leukemia and recipients of allogeneic hematopoietic stem-cell transplants (HSCT). In an autopsy-based study of 47 critically ill immunocompromised patients (including 17 with hematological malignancies) at risk for invasive aspergillosis (IA), serum BG levels were significantly higher in patients with IA than in those with no IFI [16]. However, overall performance of BG was moderate and 2 patients with bacteremia had serum BG levels higher than 140 pg/mL [16]. In a retrospective study, BG increased the rate of IA detection compared to galactomannan [13]. Importantly, BG was positive in all patients with *Pneumocystis jirovecii* pneumonia and in 85% of those with fungal bloodstream infections [13]. A metaanalysis that excluded patients with *P. jirovecii* infection showed a sensitivity of 76.8% (95% confidence interval, [95% CI], 67.1%–84.3%) and a specificity of 85.3% (95% CI, 79.6–89.7) [17]. Another metaanalysis focused on patients with hematological malignancies (*n =* 1771, including 414 with IFIs) and showed that two consecutive positive BG assays had very good specificity (98.9%; 95%CI, 97.4%–99.5%), positive predictive value (PPV, 83.5% for a prevalence of 10%), and negative predictive value (NPV, 94.6% for a prevalence of 10%) for proven or probable IFI. However, sensitivity was low (49.6%; 95% CI, 34.0%–65.3%) [18]. Performance was similar across the different BG assays [18].

No study has evaluated how BG contributes to the diagnosis of IFI in unselected patients with hematological malignancies admitted to the ICU. These patients usually have organ dysfunctions, which may affect BG performance; [4] and bacterial infections, which complicate the interpretation of BG assay results [13, 16]. Furthermore, over 90% of critically ill hematology patients receive antibacterial agents and up to 50% antifungal agents, which have been reported to modify BG values [19].

The objective of this multicenter database study conducted by the *Groupe de Recherche en Réanimation respiratoire Onco-Hématologique* (Grrr-OH) was to assess the accuracy of BG for diagnosing IFI in unselected critically ill patients with hematological malignancies.

## RESULTS

### Patients

**Figure 1:**
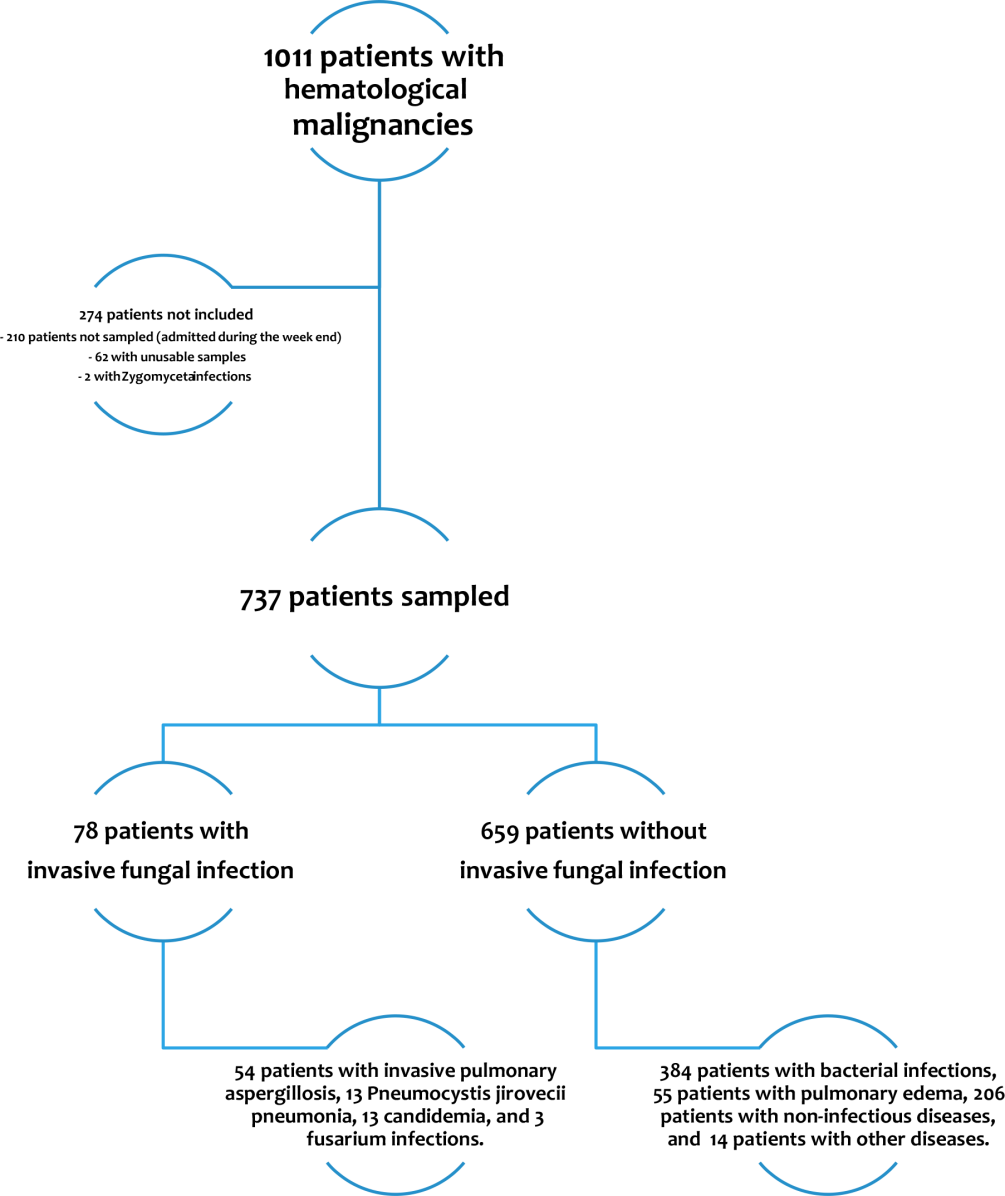
Patient flowchart (5 patients had both candidemia and another IFI)

As shown in Figure 1, of 1011 patients with hematological malignancies admitted to the study ICUs, 801 were admitted on weekdays and 737 were included in the study. These 737 patients showed no significant differences with the 274 patients who were not included. Two patients with *Zygomycetes* infections were not included.

Table 1 reports the main patient characteristics. Acute leukemia and non-Hodgkin lymphoma were the most common malignancies and 80% of patients had received chemotherapy in the past week, whereas only 174 (23.6%) were in partial or complete remission. The malignancy had been diagnosed in the past 2 weeks in 283 (38.4%) patients. Of the 184 (25%) bone-marrow transplant (BMT) or HSCT recipients, 81 had received autologous and 103 allogeneic transplants. Neutropenia at ICU admission was found in 208 (28%) patients. The main reason for ICU admission was acute respiratory failure (*n =* 398, 54%), and mechanical ventilation was provided to 439 (60%) patients. Hospital mortality was 37%.

**Table 1:** Patient characteristics at ICU admission

	IFI *n* = 78	No IFI *n* = 659	*P* value
**Age**	58 (48–64)	61 (50–70)	0.08
**Male gender**	46 (59.0%)	397 (60.2%)	0.83
**Underlying malignancy**		209 (31.7%)	0.37
**Non-Hodgkin lymphoma**	23 (29.5%)	180 (27.3%)	
**Acute myeloid leukemia**	20 (25.6%)	90 (13.7%)	
**Myeloma**	7 (9.0%)	40 (6.1%)	
**Acute lymphocytic leukemia**	9 (11.5%)	57 (8.6%)	
**Chronic lymphocytic leukemia**	5 (6.4%)	13 (2.0%)	
**Chronic myeloid leukemia**	2 (2.6%)	20 (3.0%)	
**Myelodysplastic syndrome**	1 (1.3%)	14 (2.1%)	
**Hodgkin lymphoma**	3 (3.8%)	36 (5.5%)	
**Other malignancies**	8 (10.3%)		
**Newly diagnosed malignancy ¥**	22 (28.2%)	261 (39.6%)	0.06
**Partial or complete remission**	19 (24.4%)	155 (23.5%)	0.88
**BMT / HSCT**			0.02
**No**	49 (62.8%)	504 (76.5%)	
**Autologous**	11 (14.1%)	70 (10.6%)	
**Allogeneic**	18 (23.1%)	85 (12.9%)	
**Chemotherapy in the last 7 days**	72 (92.3%)	519 (78.8%)	0.004
**Neutropenia**	34 (43.6%)	174 (26.4%)	0.002
**Reasons for ICU admission £**			
**Acute respiratory failure**	69 (88.5%)	329 (49.9%)	< 0.0001
**Shock**	52 (66.7%)	308 (46.7%)	0.001
**Acute renal failure**	9 (11.5%)	146 (22.2%)	0.03
**Sepsis**	78 (100%)	398 (60.4%)	< 0.0001
**Systemic antifungal agents**	59 (76%)	224 (34%)	< 0.0001
**Life-sustaining therapies**			
**Mechanical ventilation (invasive or noninvasive)**	65 (83.3%)	374 (56.8%)	0.02
**Vasopressors**	52 (66.7%)	308 (46.7%)	0.001
**Renal replacement therapy**	22 (28.6%)	167 (26.0%)	0.73
**Outcomes**			
**Length of ICU stay**	9 (5–17)	5 (3–10)	< 0.0001
**ICU mortality**	32 (41.0%)	158 (24.0%)	0.002
**Length of hospital stay**	9 (5–17)	5 (3–10)	< 0.0001
**Hospital mortality**	42 (53.8%)	231 (35.1%)	0.002
**BG concentrations (pg/mL) were higher in patients with than without IFI (144 (77–510) vs. 50 (30–125), < 0.0001)**	144 (77–510)	50 (30–125)	< 0.0001

¥ Hematological malignancy diagnosed in the past 2 weeks.

£ Some patients were admitted for more than one reason. IFI, invasive fungal infection; BMT, bone marrow transplant; HSCT, hematopoietic stem-cell transplant; ICU, intensive care unit; BG, (1–3)-β-D-glucan.

IFIs were documented in 78 (10.6%) patients, including 54 with IPA (5 proven, 31 probable, and 18 possible), 13 with proven *P. jirovecii* pneumonia, 13 with candidemia, and 3 with fusarium infections; 5 patients had both candidemia and another IFI. Mortality in patients without IFI was 35.1%, rising to 64.8% in patients with invasive aspergillosis (OR 3.41 (1.91–6.10)).

### (1–3)-β-D-glucan (BG) assay results

**Figure 2:**
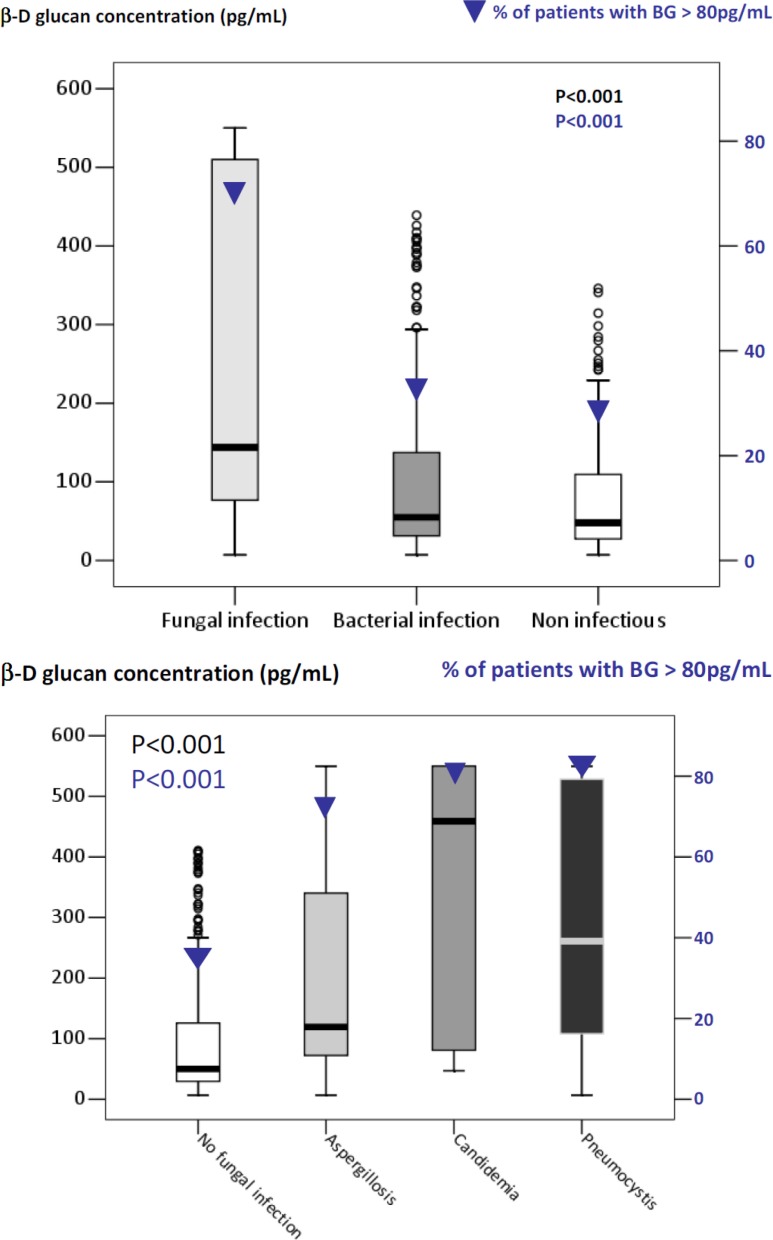
β-D-glucan concentrations (box plots, Y axis on the left) and proportion of patients with β-D-gluca*n* > 80 pg/mL (▼, Y axis on the right) according to final diagnoses (**A**) β-D-glucan was > 80 pg/mL not only in 74.4% of patients with invasive fungal infections, but also in 38.3% with bacterial infections and 30.7% with noninfectious diseases (*P* < 0.0001). (**B**) β-D-glucan was < 80 pg/mL in 2 patients with *Pneumocystis* pneumonia, 3 patients with candidemia, and 30% of patients with invasive pulmonary aspergillosis. On the opposite, β-D-glucan was > 80 pg/mL in 30% of patients without fungal infections. Patients with Fusarium infection had β-D-glucan concentrations of 76 pg/mL [75–77].

Median (25th–75th) BG concentration was 62 (37–120) pg/mL, and 32% of patients had BG concentrations above the manufacturer's cutoff (80 pg/mL). BG concentrations were higher in patients with than without IFI (144 [77–510] vs. 50 [30–125], P < 0.0001). Of the 78 patients with IFIs, 74.4% had BG > 80 pg/mL (Figure 2A). However, BG was also > 80 pg/mL in 38.3% of patients with bacterial infections and 30.7% with noninfectious diseases (*P* < 0.0001). Figure 2B shows BG results according to type of IFI. BG was < 80 pg/mL in 2 patients with *Pneumocystis* pneumonia, 3 patients with candidemia, and 30% of patients with IPA. BG was > 80 pg/mL in 35% of patients without IFIs.

### Diagnostic performance of the (1–3)-β-D-glucan (BG) assay

**Figure 3:**
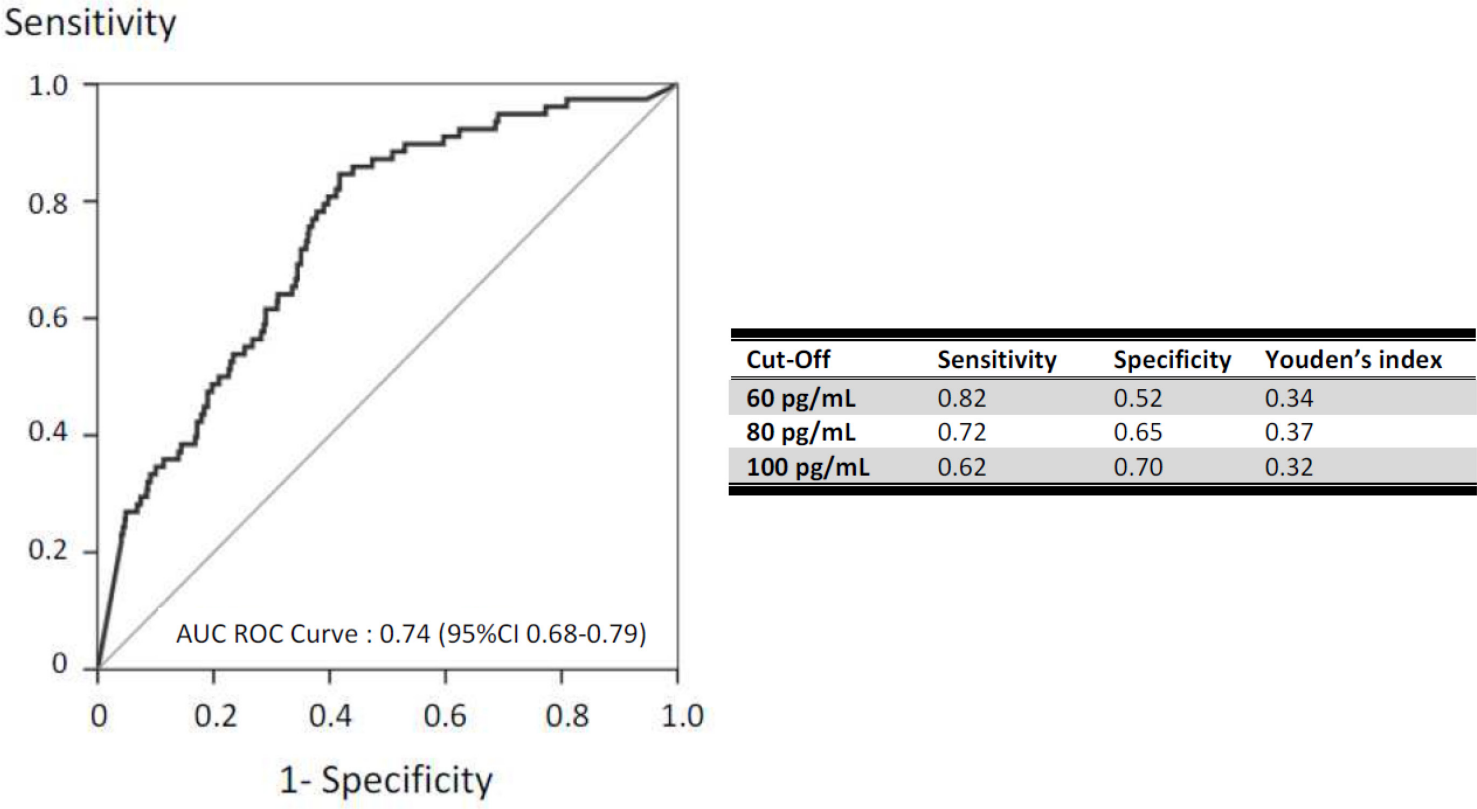
Performance of (1–3)-β-D-glucan for the diagnosis of invasive fungal infection (area under the ROC curve according to BG value) Sensitivity and sensitivity are reported for (1–3)-β-D-glucan cutoffs of 60 pg/mL, 80 pg/mL, and 100 pg/mL. The best cutoff was 80 pg/mL.

Figure 3 displays the performance characteristics of the BG assay. The manufacturer's cutoff of 80 pg/mL was associated with the best performance, with 72% sensitivity, 65% specificity, and a ROC-AUC of 0.74 (0.68–0.79). The sensitivity analyses showed no differences in performance characteristics in patients with IA (Supplementary Figure S1) or with acute respiratory failure, or allogeneic BMT/HSCT (data not shown). Additional sensitivity analyses were performed to assess the performance of a BDG > 80 pg/ml in different subgroups. It did not disclose any difference patients without neutropenia (AUC *=* 0.752 (0.678–0.826)), those not needing mechanical ventilation (AUC 0.778 (0.693–0.862)), or vasopressors (AUC 0.796 (0.715–0.877)).

Furthermore, antifungal treatment at ICU admission did not affect BG assay performance (ROC-AUCs: all patients, 0.74 [0.68–0.79]; patients without antifungal therapy, 0.72 [0.61–0.83]; and patients with antifungal therapy, 0.73 [0.66–0.80]). With a 10% prevalence of IFI, NPV was 94% and PPV 21% (Supplementary Figure S2). The contribution of a BG > 80 pg/mL to estimating the pre-test probability of IFI was fair. For instance, a 40% pre-test probability of IFI was increased to 58% by a positive BG assay and decreased to 22% by a negative BG assay (Supplementary Table S1 and Supplementary Figure S3).

### Factors affecting (1–3)-β-D-glucan (BG) assay results

Table 2 reports the multivariable analysis of factors independently associated with a positive BG (> 80 pg/mL). IFI was associated with a positive BG (OR, 4.42 [95%CI, 2.57–7.75]), as well as admission SOFA score, autologous BMT/HSCT, and microbiologically documented bacterial infection. When neutropenia was forced into the final model, this variable was not selected and did not change the results.

**Table 2:** Multivariable analysis of factors associated with a (1–3)-β-D-glucan concentration > 80 pg/mL

Variable	Odds ratio	95% confidence interval	*P* value
**SOFA score at ICU admission**	1.06 per point	1.02–1.11	0.009
**Autologous BMT/HSCT**	1.56	1.07–2.29	0.02
**Invasive fungal infection**	4.42	2.57–7.75	< 0.0001
**Documented bacterial infection**	1.41	0.96–2.07	0.08

Hossmer Lemeshow Chi-square: 11.07; df = 8, *P* = 0.20.

### Estimated clinical impact of the (1–3)-β-D-glucan (BG) assay

**Figure 4:**
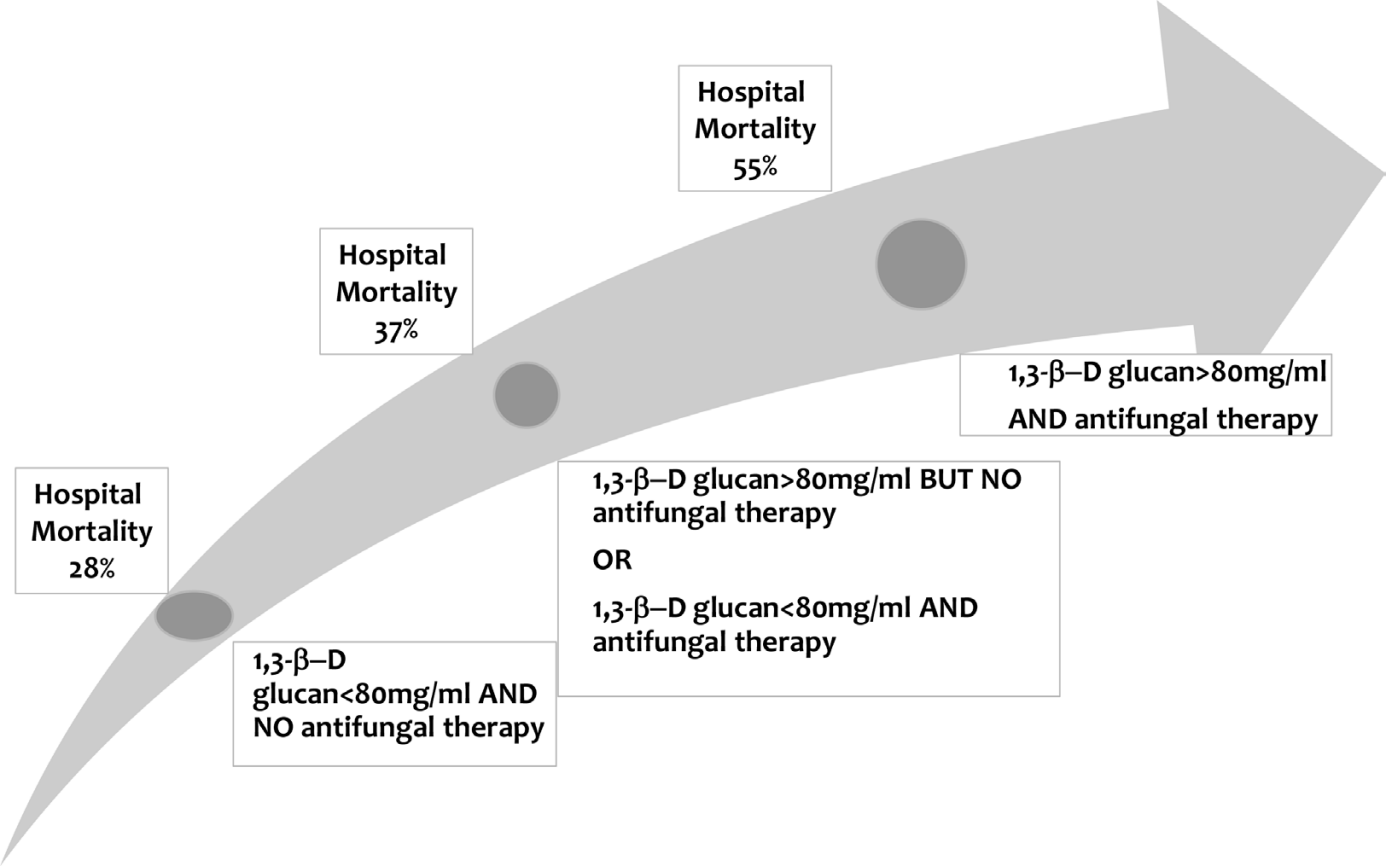
Hospital mortality according to (1–3)-β-D-glucan concentrations and antifungal therapy in the ICU *P* < 0.0001 across the four groups.

Among the 78 patients with a final diagnosis of IFI, only 59 (76%) received systemic antifungals during the ICU stay; thus, 19 (24%) patients with IFI were left untreated. Had the BG assay results been communicated to the managing physicians and treatment given when (and only when) BG was > 80 pg/mL, then 12 of these 19 patients would have received antifungals but 15 of the 59 treated patients with IFIs would have had their antifungals withdrawn. Among the 659 patients without IFI, 145 would have received antifungal therapy and 135 would have had their antifungal therapy withdrawn. Figure 4 reports the relation between hospital mortality, BG concentrations, and antifungal therapy. Antifungal agents were administered to 46% of patients with BG > 80 pg/mL and to 33% of patients with BG < 80 pg/mL. Figure 4 shows significant differences in hospital mortality across patient groups defined by BG concentrations and use of systemic antifungals.

## DISCUSSION

The (1, 3)-β-D-glucan assay has been recommended for diagnosing IFIs in high-risk patients with hematological malignancies [1]. This assay has been used to guide clinical management decisions in conjunction with clinical, microbiological, and radiological findings [8]. However, the diagnostic accuracy and optimal cutoff of the BG assay in unselected critically ill hematology patients remain unclear. We evaluated these two points in a population that reflects everyday clinical practice. Furthermore, our sample size was sufficiently large to allow separate assessments of diagnostic accuracy in high-risk patients (i.e., patients with acute leukemia and prolonged neutropenia after induction/consolidation chemotherapy or allogeneic HSCT) and in various types of fungal infections.

Many of the factors reported to impair the performance of the BG assay are frequently present in critically ill patients. False-negative BG results occur in patients receiving antifungal agents and in those with IFIs due to organisms not detected by the BG assay [10]. However, in our study, BG concentrations were not affected by prophylactic or curative antifungal therapy. Moreover, we did not include patients with IFI due to *Zygomycetes*, which is not detected by the BG assay. Factors associated with false-positive BG results include blood transfusions or administration of blood products [1] (70% of our patients), renal replacement therapy [7] (27%), β-lactam antibiotics (90%) and bacterial infection (53%) [26]. These factors may explain the limited sensitivity and specificity of the BG assay in critically ill hematology patients in our study.

The contribution of the BG assay to the diagnosis of IFI in our population was modest: sensitivity and PPV were low, and the increase in pre-test probability of IFI was moderate. However, the good NPV of the BG assay suggests a possible role in deciding antifungal agent de-escalation in high-risk patients with low pre-test probability. With antibacterial agents, de-escalation has been found feasible and safe in general ICU patients [27, 28] and in ICU patients with neutropenia [29]. Fungal infections are associated with high morbidity and mortality rates in hematology patients. Establishing a definitive diagnosis of IFI is difficult. The signs and symptoms are nonspecific and may not develop until the disease is advanced or disseminated. Antifungal agents are therefore given prophylactically or pre-emptively in high-risk patients. Withdrawing unnecessary antifungal therapy decreases the risk of toxicity and drug interactions and diminishes healthcare costs. Studies are needed to determine whether the BG assay is effective and safe in guiding decisions to de-escalate antifungal therapy in ICU patients with hematological malignancies.

In our study, among patients with BG > 80 pg/mL who received antifungal agents, 55% died. This rate is significantly higher than mortality rates in patients with either low BG or no antifungal therapy but is nevertheless considerably lower than previously reported in critically ill hematology patients with IFIs. Overall mortality in patients with IFIs was 53.8%. This suggests that the physicians in the 17 participating ICUs, who were blinded to BG results, maintained a high level of suspicion regarding IFI. This possibility is consistent with an autopsy study showing that the proportion of patients who received antifungal agents but had no IFI was larger than the proportion who died from untreated IFI [30]. This point further supports the need for studies of antifungal agent withdrawal in patients with no proof of IFI and low BG levels.

This study has several limitations. First, BG was assayed only once, at ICU admission. Serial measurements of fungal cell-wall biomarkers may be more effective both for diagnosing IFI and for assessing the therapeutic response. However, this study is the first to assess the diagnostic accuracy of the BG assay in a large cohort of critically ill hematology patients. Second, we did not focus on a single fungal infection. However, we analyzed the patients with IA separately and found that the performance of the BG assay in this subgroup was similar to that in the overall population. Nevertheless, whereas the BG assay was previously reported to be consistently positive in patients with *P. jirovecii* pneumonia, 2 such patients had a negative BG assay in our study. A careful assessment of pre-test probabilities of each etiology is essential, and the BG assay should be interpreted in the light of a broad array of clinical, laboratory, and imaging findings. Last, etiological diagnoses were made by consensus among three independent experts, based on the most recent diagnostic criteria for IFI. However, these criteria may lack sensitivity, and some patients with high BG concentrations who were classified as not having IFI may have had undiagnosed IFI. In patients with febrile neutropenia and lung infiltrates, combining multiple diagnostic tests, including PCR assays, provided only modest increases in the sensitivity and PPV predictive value of the BG assay [19]. Moreover, BG levels were higher in patients with IFI proven by autopsy than in patients without IFI [16].

In conclusion, the diagnostic usefulness of BG concentrations in critically ill patients with hematological malignancies is limited by low sensitivity and specificity. However, the good NPV may warrant studies to assess the usefulness of the BG assay for identifying patients whose antifungal treatment can be safely withdrawn.

## PATIENTS AND METHODS

### Patients

We studied patients from the Grrr-OH database described in a previous publication [4]. Consecutive adults (> 18 years) with hematological malignancies admitted to 17 ICUs in France and Belgium over a 16-month period in 2010–2011 were included into the database (ClinicalTrials.gov Identifier: NCT01172132). As described previously, [4] we excluded patients who had been disease-free for more than 5 years or were admitted to the ICU only to undergo a procedure under optimal safety conditions. The study was approved by the appropriate ethics committees in France and Belgium. All patients or relatives gave their informed consent to study participation.

### Data collection

In each center, an investigator recorded the study data on a standardized electronic case-report form. In all 17 centers, a senior intensivist and a senior hematologist were available around the clock and made decisions together. All diagnostic strategies and antifungal treatment decisions were based on clinical judgment, according to standard practice in the Grrr-OH research group.

The data in the tables and figures were collected prospectively. The Sepsis-Related Organ Failure Assessment (SOFA) score was computed at admission [20] to estimate the risk of death based on organ dysfunctions. Reasons for ICU admission were recorded based on the main symptoms at ICU admission [21]. Acute respiratory failure was defined as previously reported [22, 23] and neutropenia as a neutrophil count < 500/mm^3^ [24]. Prespecified criteria were applied to define the causes of pulmonary involvement; [5] for possible or probable IA, the most recent definitions were used [1]. Candidemia was defined as recovery of any *Candida* species from at least one blood culture [25]. Invasive fusarium infections were documented by blood culture or skin biopsy. Confirmed *Pneumocystis* pneumonia was presence of *P. jirovecii* by Gomori-Grocott or toluidine-blue stain, or a positive immunofluorescence test, on bronchoalveolar lavage (BAL) fluid or induced sputum. Mucormycoses were documented as previously described [1].

### Etiological diagnosis

Etiologic diagnoses were these that required the need for ICU admission and present at the time the patients was sampled for BG dosage. They were made by consensus among the managing physicians (intensivists, hematologists, and consultants), according to recent definitions [4, 5]. Only IFIs present at ICU admission or in the first 5 days of ICU admission were considered. For the study, the diagnosis in each patient was reevaluated by three independent experienced intensivists (EA, FV, VL) who were blinded to the initial diagnosis and had access to all the clinical and microbiological data. They disagreed with the managing physicians for 87 patients, 70 with non-fungal etiologies and 17 with IFIs. Of these 17 patients, 6 had microbiological samples that became positive after their death (3 candidemia and 3 *Aspergillus* in the lower respiratory tract), 5 were reclassified as having probable instead of possible invasive pulmonary aspergillosis [IPA], 2 with a PCR-based diagnosis of *Pneumocystis* pneumonia were reclassified as having *Pneumocystis* colonization, and 4 with possible IPA were reclassified as having no identifiable cause (2 had pulmonary findings consistent with leukemic infiltrates but no CT scan and were quickly discharged from the ICU and 2 had normal CT scans). These disagreements were resolved by consensus between the main investigator and the investigator at the relevant study center.

### (1–3)-β-D-glucan assay

All patients admitted on weekdays had an arterial and/or venous blood sample taken at admission for a BG assay. The serum was processed within 6 h of collection then stored at −20°C until batch testing was performed. The BG assay Fungitell^®^ (Associates of Cape Cod Inc., Falmouth, MA, USA) was used as recommended by the manufacturer, with the ELx808^™^ Absorbance Microplate Reader and Gen5^™^ Data Analysis Software (Biotek Instruments, Winooski, VT, USA). The BG concentration in each sample was calculated automatically using a calibration curve established from standard solutions containing 31.25 pg/mL to 500 pg/mM of BG. We evaluated the manufacturer-recommended BG cutoff of 80 pg/mL and other cutoff values. All materials used for the assay were glucan-free. Each sample was assayed in triplicate, and the mean of the three values was used for the study. When one of the three values differed markedly from the other two, it was discarded, provided the other two results had a coefficient of variation ≤ 20% for BG concentrations below 200 pg/mL; for higher BG concentrations, the assay was repeated if the coefficient of variation was > 100%. The assay results were not reported to the managing physicians.

### Statistical analysis

Continuous variables were described as medians (25th–75th quartiles) and compared using the Mann-Whitney *U* test. Categorical variables were described as percentages and compared using the two-tailed χ^2^ test or Fisher's exact test, as appropriate. The following parameters of diagnostic performance and their 95%CIs were calculated: sensitivity, specificity, PPV, NPV, and Cohen's kappa. The discriminatory power of BG was evaluated by computing areas under the receiver-operating characteristic curves (ROC-AUCs), which were compared as described by Hanley and McNeil.

We performed logistic regression analyses to identify variables significantly associated with BG concentrations > 80 pg/mL, by estimating the odds ratio (OR) with the 95%CI. Variables yielding *P* values lower than 0.20 by univariate analyses or considered clinically relevant were entered into a backward stepwise logistic regression model. We checked that continuous variables were log-linear. The covariates were entered into the model with critical entry and removal *P* values of 0.20 and 0.1, respectively. Multicollinearity and interactions were tested. The Hosmer-Lemeshow test was used to check goodness-of-fit of the logistic regression model. Last, because neutropenia is a major clinical risk factor and confounder for fungal infection in patients with hematological malignancies, the predefined statistical analysis plan involved forcing this variable into the final model in the event it was not selected. All tests were two-sided, and *P* values lower than 0.05 were considered statistically significant. Statistical tests were done using the SPSS 13 software package (IBM, Armonk, NY, USA).

## SUPPLEMENTARY MATERIALS TABLE AND FIGURES



## Abbreviations list

95%CI: 95% confidence interval
AUC: area under the curve
BAL: bronchoalveolar lavage
BG: (1–3)-β-D-glucan
BMT: bone marrow transplant
CT: computed tomography
EORTC: European Organization for Research and Treatment of Cancer Mycoses Study Group
Grrr-OH: *Groupe de Recherche en Réanimation respiratoire Onco-Hématologique* (oncology-hematology respiratory research group)
HM: hematological malignancies
HSCT: hematopoietic stem-cell transplant
ICU: intensive care unit
IFI: invasive fungal infection
MSG: Mycoses Study Group
NPV: negative predictive value
OR: odds ratio
PCR: polymerase reaction chain
PPV: positive predictive value
ROC: receiver-operating characteristic
SOFA: Sequential Organ Failure Assessment
