# Supplementary material for: (1, 3)-β-D-glucan assay for diagnosing invasive fungal infections in critically ill patients with hematological malignancies
Source: Oncotarget. 2016 Feb 18;7(16):21484–95. doi: 10.18632/oncotarget.7471 (PMC5008300; doi:10.18632/oncotarget.7471)
Supplement: Supplementary file 1 [file oncotarget-07-21484-s001.pdf]

(1, 3)-β-D-glucan assay for diagnosing invasive fungal infections in critically ill patients with hematological malignancies

Supplementary Materials

Supplemental Table S1: Fungal infection probability according to pre-test probability and (1–3)-β-D-glucan value

Pre-test probability	Post-test if BG > 80 pg/mL[a]	Post-test if BG < 80 pg/mL[a]
**20%**	34%	10%
**40%**	58%	22%
**50%**	67%	30%
**60%**	76%	39%
**80%**	89%	63%

BG, (1–3)-β-D-glucan value.
[a]80 pg/mL was the optimal BG cutoff.

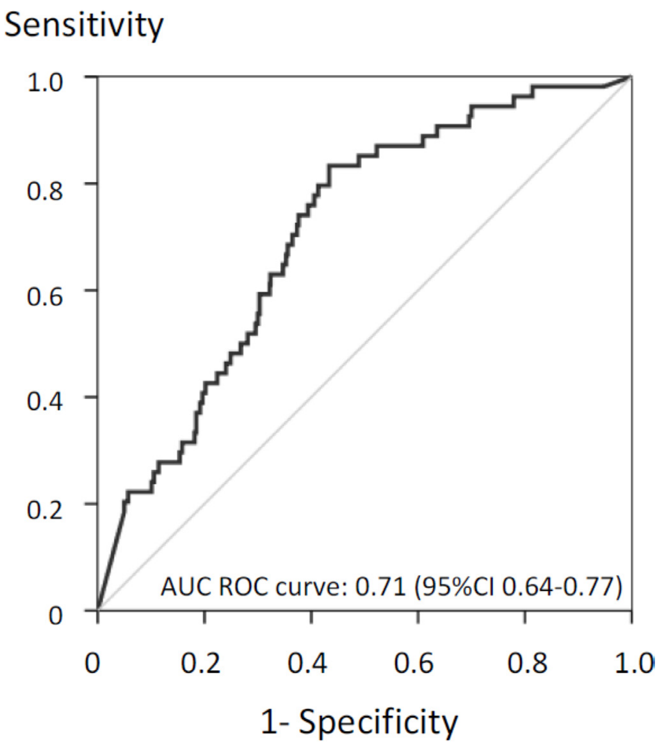

Supplemental Figure S1: Performance of the (1–3)-β-D-glucan (BG) assay for diagnosing invasive aspergillosis (area under the ROC Curve according to BG value).

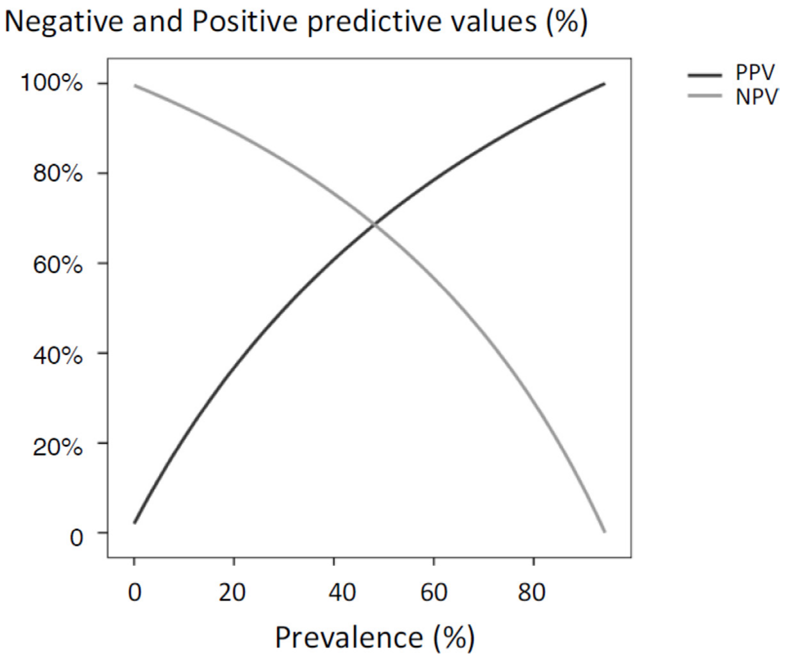

Supplemental Figure S2: Changes in positive predictive value (PPV) and negative predictive value (NPV) of the (1–3)-β-D-glucan (BG) assay interpreted as positive when ≥ 80 pg/mL according to the prevalence of invasive fungal infection.

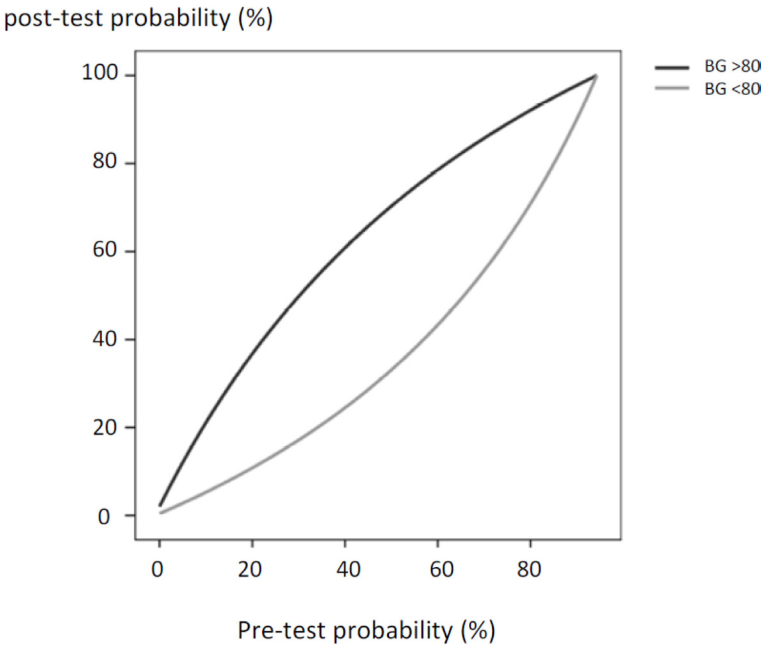

Supplemental Figure S3: Pre- and post-test probabilities of invasive aspergillosis according to (1–3)-β-D-glucan (BG) assay results.
